# Endobronchial Obstruction: A Case of Well-Differentiated Liposarcoma

**DOI:** 10.7759/cureus.57731

**Published:** 2024-04-06

**Authors:** Enrique F Martinez Trevino, Momena Sohail, Ivan A Mijares-Rojas, Seba Qaddorah, Anne Levenson

**Affiliations:** 1 Internal Medicine, John H. Stroger, Jr. Hospital of Cook County, Chicago, USA; 2 Pulmonary and Critical Care Medicine, John H. Stroger, Jr. Hospital of Cook County, Chicago, USA; 3 Internal Medicine, University of Miami, Jackson Memorial Hospital, Miami, USA

**Keywords:** cryoablation, well-differentiated liposarcoma, liposarcoma, flexible bronchoscopy, endobronchial tumor

## Abstract

Atypical lipomatous tumor/well-differentiated liposarcoma (ALT/WDL) is a rare subtype of endobronchial tumor that has been rarely reported in medical literature. Due to its low incidence, distinguishing it from endobronchial lipoma poses a significant diagnostic challenge, necessitating histopathologic and cytogenetic analysis. As of today, the treatment and surveillance protocols for these neoplasms remain poorly defined, often resulting in their misclassification and treatment as endobronchial lipomas. We present a case involving a 72-year-old male who presented with worsening dyspnea and cough. Diagnostic imaging revealed an endobronchial lesion obstructing the left main bronchus. The patient underwent a flexible bronchoscopy that identified a polypoid mass causing significant obstruction, which was subsequently resected via cryoablation. Histopathology confirmed ALT/WDL, supported by genetic analysis revealing chromosomal alterations. Following the intervention, symptoms resolved, with no recurrence on follow-up imaging. Differentiating ALT/WDL from endobronchial lipomas is necessary not only because it influences treatment decisions but also because it can significantly affect the prognosis of patients diagnosed with ALT/WDL. In this case, we emphasize the importance of considering ALT/WDL in the differential diagnosis of endobronchial tumors and highlight the use of flexible bronchoscopy as a viable substitute for rigid bronchoscopy, serving not only as a diagnostic tool but also as a therapeutic method.

## Introduction

Atypical lipomatous tumor/well-differentiated liposarcoma (ALT/WDL) is an exceptionally rare subtype of endobronchial tumor that has rarely been described in medical literature. These tumors are part of the group of primary endobronchial neoplasms, which account for 0.6% of all lung neoplasms [1]. Because of their low incidence, the clinical presentation and management of ALT/WDL remain poorly defined, and most of the available knowledge is derived from cases of endobronchial lipomas. Benign endobronchial tumors such as endobronchial lipomas show a strikingly similar clinical presentation to ALT/WDL, and differentiation is achieved by specific cytogenetic molecular analyses. In an effort to advance our understanding of endobronchial well-differentiated liposarcomas, we present the case of a male in his 70s with an endobronchial ALT/WDL who presented with symptoms indicative of airway obstruction and was successfully treated via flexible bronchoscopy and cryotherapy.

## Case presentation

A 72-year-old male with a medical history of hypertension, type 2 diabetes mellitus, hyperlipidemia, and chronic bilateral venous insufficiency was admitted to the hospital due to worsening dyspnea on exertion and cough that had been ongoing for six weeks. The patient reported experiencing shortness of breath after minimal physical activity, such as climbing stairs, accompanied by a productive cough with greenish sputum, primarily at night. Notably, his breathing improved after clearing the phlegm. He denied having fever, chest pain, orthopnea, or paroxysmal nocturnal dyspnea but did mention chronic swelling in both lower extremities and a weight loss of ten pounds over one month.

**Figure 1:**
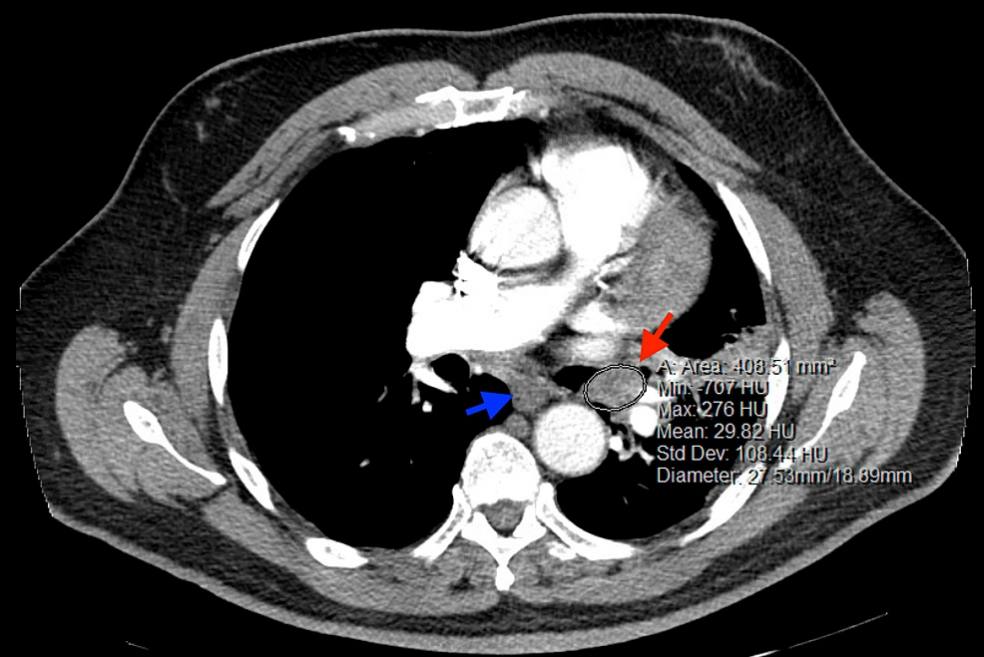
CT chest (axial view) demonstrating the 2.7 x 1.9 cm ovoid mass in the left main-stem bronchus (red arrow) and enlarged mediastinal soft tissue (blue arrow)

Upon admission, the patient's vital signs were stable, and his oxygen saturation level was 97% on room air. Physical examination revealed reduced air entry and breath sounds in the left mid and lower lung lobes. Initial blood work, including the complete blood count, basic metabolic panel, venous blood gases, and coagulation panel, showed normal results. Although the chest X-ray did not provide a definitive diagnosis, it indicated opacification of the left lower lobe (LLL). A CT angiogram of the chest was performed, revealing a 2.7 x 1.9 cm mass in the left main-stem bronchus, causing partial obstruction of the LLL bronchi and resulting in partial collapse and enlargement of the lymph nodes in the mediastinum (Figure 1).

**Figure 2:**
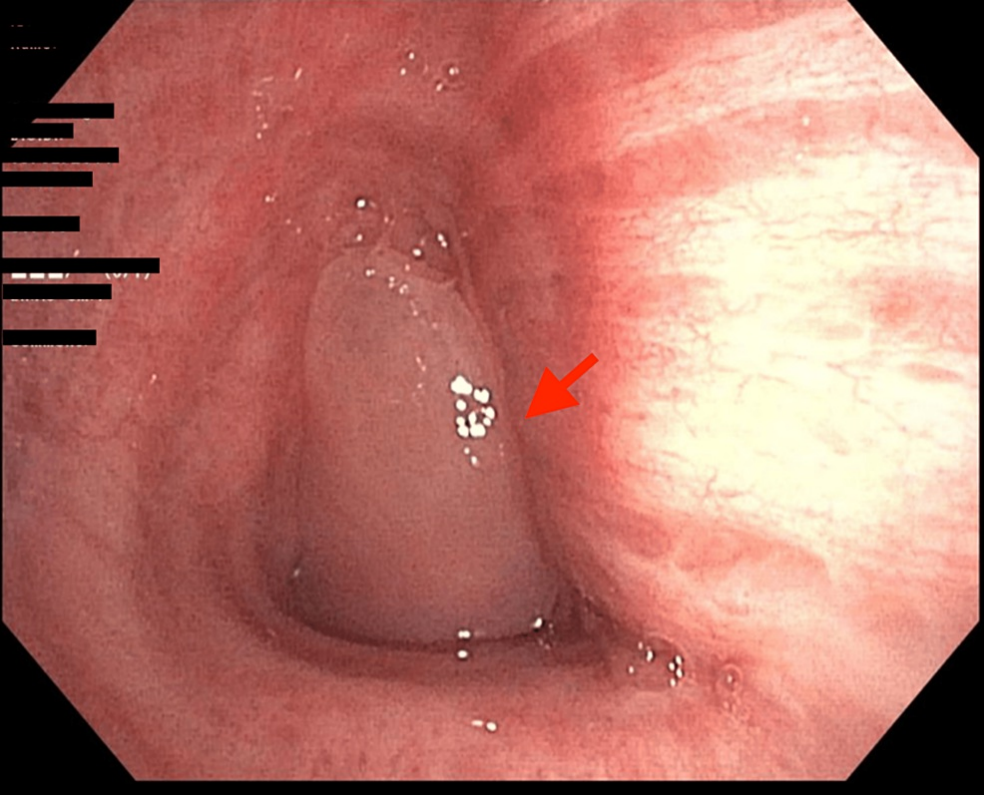
Bronchoscopic view of the left main stem bronchi showing an endobronchial mass causing 90% obstruction (red arrow)

The patient initially underwent a flexible bronchoscopy, during which a mobile, whitish polypoid mass was observed, causing 90% obstruction of the left distal main bronchus (Figure 2). However, due to inadequate tissue samples, initial biopsies obtained through this initial intervention did not confirm a malignant pathology.

**Figure 3:**
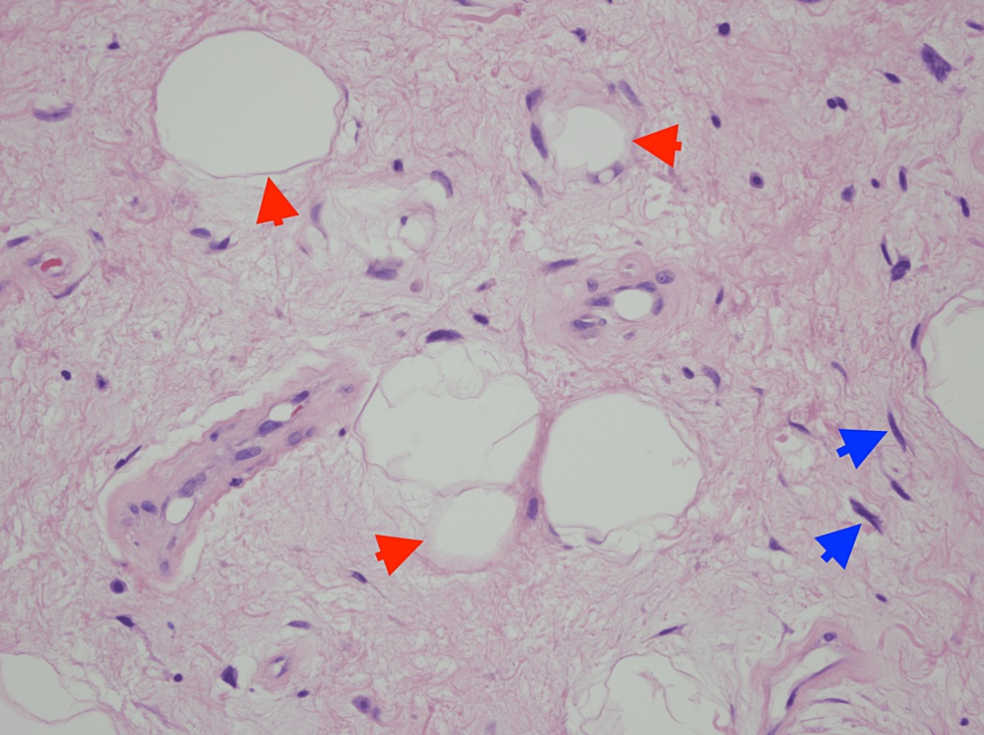
Variably sized, mature adipocytes (red arrows) and bands of fibrotic stroma containing spindle cells with enlarged, hyperchromatic nuclei (blue arrows)

Therefore, the patient underwent a subsequent flexible bronchoscopy procedure with en-bloc resection and cryoablation. Histopathologic analysis of the removed tissue indicated the presence of mature adipocytes within a fibrotic background, along with atypical spindle cells suggestive of well-differentiated liposarcoma (Figure 3).

Genetic analysis also revealed multiple chromosomal alterations, including gains and losses in various chromosomal regions, but no alterations in the MDM2 or CDK4 genes were observed. An attempted fluorescence in situ hybridization (FISH) analysis failed due to hybridization issues. The final pathologic diagnosis was well-differentiated liposarcoma.

After the intervention, the patient's symptoms improved, and he no longer experienced dyspnea or a productive cough. A follow-up chest X-ray performed one month after resection of the mass revealed improved ventilation in the LLL with no evidence of recurrence. The follow-up plan included periodic monitoring, with a chest CT scan scheduled every four months for two years and a bronchoscopy scheduled in one year for surveillance purposes.

## Discussion

Liposarcomas account for approximately 20% of all new soft tissue sarcoma diagnoses [2]. These tumors are classified based on their histopathological characteristics, including dedifferentiated liposarcoma, myxoid cell liposarcoma, pleomorphic liposarcoma, rare mixed-type liposarcoma, and ALT/WDL [3]. They are typically found in the lower extremities (75%) or the retroperitoneum (20%) [4], although cases of endobronchial, mediastinal, or esophageal liposarcomas have also been reported [4]. Primary endobronchial ALT/WDL exhibits aggressive local growth with a low likelihood of recurrence and a low risk of metastasis [3]. Early resection and treatment prevent airway disease and obstructive complications such as post-obstructive pneumonia, bronchiectasis, hemoptysis, or empyema [5,6].

Most endobronchial lipomatous tumors present with symptoms at the time of diagnosis; however, due to their low incidence, they are often misdiagnosed as asthma or chronic bronchitis and can remain undetected for months or years [7]. In a 2003 case series, Muraoka et al. reported 64 cases of endobronchial lipoma, where they found that 48 patients (75%) were symptomatic at the time of diagnosis, presenting with symptoms such as cough (40 cases), expectoration (18 cases), hemoptysis (seven cases), elevated temperature (10 cases), and dyspnea (six cases). The remaining 25% did not show any symptoms at the time of diagnosis [8]. On the other hand, ALT/WDL clinical presentations have been rarely reported. In 2018, Liu et al. described for the first time a case of ALT/WDL in a patient in his 50s with involvement of the left main bronchus. The patient presented with a one-year history of cough, left-sided chest pain, and worsening dyspnea on exertion [9]. In our case, the patient presented with a productive cough and worsening exertional dyspnea, which are characteristic symptoms of endobronchial lipomatous tumors as previously described. What distinguished our case was the improvement of dyspnea after expectoration, suggesting a temporary total obstruction relieved by clearing the phlegm.

It is important to acknowledge that distinguishing between benign endobronchial lipomas and primary endobronchial ALT/WDL presents a complex challenge due to their closely resembling presentations and shared characteristics. Employing genetic analysis and utilizing FISH to identify specific genetic markers has emerged as a valuable strategy for early differentiation. This early classification not only influences treatment decisions but also significantly impacts the overall prognosis of individuals diagnosed with endobronchial liposarcomas. Approximately 90% of ALT/WDL display chromosomal amplification in the 12q13-15 region, leading to overexpression of the oncogenes MDM2 and CDK4 [9]. In contrast, endobronchial lipomas are characterized by the expression of HMGA2 and HMGA1 [3]. Identifying these molecular markers plays a crucial role in distinguishing ALT/WDL from other types of endobronchial lipomatous tumors and other types of endobronchial liposarcomas.

Endobronchial lesions are typically reported as nonspecific findings on chest X-rays. Therefore, chest CT prevails as the optimal non-invasive method for evaluating tracheal and main bronchial lesions [10]. Once an endobronchial tumor is determined, invasive procedures are necessary to obtain tissue samples and the necessary information for diagnosis. Direct endobronchial visualization by bronchoscopy is the optimal approach for acquiring endobronchial tumor tissue. While flexible bronchoscopy is commonly utilized as the initial diagnostic method, its effectiveness in tissue sampling is limited due to the small size of the forceps used. The diagnostic yield of flexible bronchoscopy has been reported to be around 65% [11]. A study conducted in 2007 that included 38 cases of endobronchial lipomas, revealed that only 10 cases (26%) were diagnosed through specimens obtained during flexible bronchoscopy [6]. Conversely, using rigid bronchoscopy for tissue retrieval exhibits a significantly greater diagnostic yield of 80% [6,11,12]. Furthermore, endobronchial cryosurgery has emerged as a highly promising technique for acquiring tissue samples from endobronchial masses [13]. Notably, it can be conducted using either flexible or rigid bronchoscopy, enhancing its versatility. This method has exhibited an impressive diagnostic yield, estimated at around 98%. This high accuracy in obtaining diagnostic specimens underscores the potential of endobronchial cryosurgery as an effective tool for diagnosing and characterizing endobronchial masses [11,13]. In the case presented, an initial flexible bronchoscopy was done to obtain tissue for biopsy. However, the pathology remained non-diagnostic after this initial procedure, requiring a second intervention.

The treatment of endobronchial liposarcomas has not been standardized due to their low incidence. However, for ALT/WDL, which have a lower metastasis rate compared to other types of liposarcomas, the primary treatment approach is complete en bloc resection with clean margins using bronchoscopy or surgery. Different bronchoscopic procedures can be utilized to achieve a total reduction of endobronchial tumors, such as rigid bronchoscopy with mechanical reduction, cryotherapy, Nd-YAG laser, argon plasma coagulation (APC), and electrocautery [14]. While the efficacy of these procedures for treating ALT/WDL has not yet been fully described, in 2018, Liu et al. reported a case of successful resection of a well-differentiated endobronchial liposarcoma using APC [9]. In our case, the ALT/WDL was successfully resected via flexible bronchoscopy using cryotherapy. Further research is needed to determine the optimal approach to resection and surveillance.

## Conclusions

Our case report describes the excision of an endobronchial ALT/WDL through flexible bronchoscopy and conducts a thorough review of the literature regarding this uncommon condition. Anticipated advances are based on genetic analysis of endobronchial liposarcomas to improve the diagnostic accuracy of these tumors. This will facilitate the diagnosis of endobronchial ALT/WDL, with the end goal of providing more data for future research and promoting the development of optimal strategies for tumor management and surveillance.
